# Praying for a Miracle: Negative or Positive Impacts on Health Care?

**DOI:** 10.3389/fpsyg.2022.840851

**Published:** 2022-04-21

**Authors:** Miriam Martins Leal, Emmanuel Ifeka Nwora, Gislane Ferreira de Melo, Marta Helena Freitas

**Affiliations:** ^1^Catholic University of Brasília, Brasília, Brazil; ^2^Saint Bonaventure Institute, Affiliated to the Pontifical Saint Bonaventure (Rome), Brasília, Brazil; ^3^Psychology of Sports, Department of Psychology, Catholic University of Brasília, Brasília, Brazil; ^4^“Religion, Mental Health, and Culture” Laboratory, Catholic University of Brasília, Brasília, Brazil

**Keywords:** miracle, coping, spiritual/religious coping, prayer, qualitative research, quantitative research, phenomenological approach

## Abstract

The belief in miracle, as a modality of spiritual/religious coping (SRC) strategy in the face of stress and psychic suffering, has been discussed in psychological literature with regard to its positive or negative role on the health and well-being of patients and family members. In contemporary times, where pseudo-conflicts between religion and science should have been long overcome, there is still some tendency of interpreting belief in miracle – as the possibility of a cure granted by divine intervention, modifying the normal course of events in a bleak medical diagnosis – as having unhealthy impacts in the care and treatment of health. This position seeks to find a base in the three characteristics of hoping in a miracle, frequently pointed out by psychological literature: (a) it would imply a negation of reality instead of its confrontation; (b) it would be a coping strategy focused on emotion instead of the problem; (c) it would imply seeking to modify the supposed desire of God by extra-natural facts. In this study, we shall critically discuss this position and the dangers of its crystallization by the use of SRC scales in which the act of praying for a miracle is previously classified as a negative strategy. We revisit some tendencies in psychological literature about the subject, taking into consideration the various facets of miracle, sociocultural facts, elements of idiographic nature, and their profound outcomes in the lives of people especially in health contexts. We illustrate the dangers of a hasty generalization of the results of nomothetic studies about the role of belief in miracle with two examples of research in the Brazilian context: one carried out with pregnant women with fetal malformation and the other with family members caring for children and adolescents with cancer under chemotherapeutic treatment. In both studies, the results do not confirm the predominance of the negative aspects associated with the act of praying for a miracle, which we discuss and analyze in light of the phenomenological perspective. In this perspective, “pray for a miracle”, as experienced by patients and caregivers, can be recognized as an act of openness to life (instead of isolation in a bleak perspective), bolstering hope, and the resignification of reality in the psyche.

## Introduction

The intersection between religion and science was established in a conflicted way in the West in the Renaissance period. However, since the 1990s, there has been a burgeoning of scientific research on the theme of religiosity and spirituality (Koening, 2012) that has enhanced a rapprochement between the health sciences and these subjects. Such approximation becomes so striking in crucial circumstances of human life such as birth, chronic illness, suffering, and death (Sulmasy, 2013). In such a new phase of the interaction between religion and science, especially the health sciences, there is not necessarily a hierarchical relationship of one over the other. Religion and spirituality, in clinical practice, are the patients’ instruments of coping in diverse ways with critical situations such as the diagnoses of chronic illnesses, a majority of them being dismal prognoses, and harrowing treatments that lead to permanent sequela or even death, and as such constitute the so-called spiritual/religious coping (SRC; Pargament and Park, 1997).

On developing the concept of SRC, defined as a process whereby people, through faith, spirituality, religiosity, or religion, seek to understand and cope with significant circumstantial demands in their lives, Pargament and his collaborators (Pargament and Hahn, 1986; Pargament, 1990; Pargament et al., 1990) have made substantial contributions to the research about the role of religiosity in healthcare. One of the results of their research is the creation of a scale that seeks to quantify SRC (Pargament et al., 2000), thereby enhancing wide-ranged research of nomothetic nature in different health contexts. This scale was later translated and adapted to various languages and countries including Brazil (Panzini and Bandeira, 2005), where a significant expansion of studies in health that employ this instrument has been observed in recent years (Panzini and Bandeira, 2007; Corrêa et al., 2016; Esperandio and August, 2017; Foch et al., 2017; Huang and Torres, 2018).

Both the original definition of the concept of SRC and the elaboration (and the respective translations and adaptations) of the scale that sets out to measure it has two concerns: “the need to cope with the event itself and the need to cope with one’s feeling and behavior in relation to that event” (Pargament and Hahn, 1986, p. 196). Furthermore, considering the manner that SRC strategies are manifest, they can be classified as active or passive, focused on the problem or emotion, bearing in mind that since the original version of the scale, such strategies were also classified as positive SRC (PSRC) – when they lead to a real facing of the problem, or personal and spiritual growth, promoting satisfaction with life, even in times of crises – or as negative SRC (NSRC) – when they tend to impair physical or mental health and lead to the increase of depression, anxiety, suffering, desperation, or increase of guilt in the face of stress factor (Pargament et al., 2000).

The prospect of a miracle or the act of praying for a miracle was one of the strategies of SRC included in the scale in question through the affirmation “I prayed for a miracle.” In fact, hope in a miracle is much more frequent in health contexts (Green, 2015; Dossey, 2018; Bibler et al., 2020) and nurtured by patients, family, and sometimes by the professionals who care for them. This manner of confronting illness may pose a veritable challenge to health professionals and has been frequently described as having a negative feature that portrays a kind of shying away from reality, creating fantastic thoughts (Vasconcelos and Petean, 2009; Borges and Petean, 2018), or implies a delegating of the grappling with moments of crises outside of oneself (to the Divine, Sacred, or God) to prayer, which, according to some authors, is anchored on the wish to modify the will of God in favor of the will of the patient (Panzini, 2004). Such a perspective is based on the extra-natural concept of a miracle that infringes natural laws (Aurélio., 2010).

As we shall see later in more details in this study, in the complete version of the North American SRC scale (called RCOPE), elaborated by Pargament et al. (2000), later on translated and adapted to Brazil by Panzini and Bandeira (2005), the act of praying for a miracle was classified as a negative feature, that is to say, NSRC. However, belief in miracles still receives little attention in health research, and qualitative studies, like those carried out by Shinall et al. (2018), which tend to see not only negative aspects but also positive ones, especially in countries with a high religious population, like Brazil, where 92% of the population declare having some religion (Brasil, 2021) and with a high dynamicity of movement between diverse religions (Mota et al., 2012).

It naturally follows that the positive/negative dichotomy surrounding the act of praying for a miracle, as a modality of SRC, is full of relevant implications for clinical practice, especially in specific cultural contexts different from the North American social reality where the scale in question was originally conceived. Therefore, the aim of this study is to discuss the role of belief in a miracle as one of the SRC strategies and to problematize the dichotomy adopted by quantitative research (that employs the scale in question) carried out in Brazil. Besides exploring the various aspects of a miracle, based on the available literature on the subject, the problematization is underpinned by a phenomenological perspective and qualifies intentionality in the act of praying for a miracle. Thereby, we intend to encourage a more effective dialog between theology and psychology as well as retrieving the value of a culturally contextualized research of idiographic nature.

To this end, this study is arranged into five topics. After this short introduction, we present the concept of a miracle in the ordinary perspective (of common dictionaries) and the philosophical, phenomenological, and theological perspectives; its different psychological meanings are also discussed. This is followed by a detailed analysis of the multiple aspects that characterize the act of praying for a miracle which may determine or not its classification as negative and/or positive SRC, especially in health settings. Thenceforth, by way of illustrating the problematization presented, we bring up two examples of studies carried out in Brazil – that employed the RCOPE scale of Pargament et al. (2000), translated by Panzini and Bandeira (2005) – and their respective findings. Finally, a discussion that integrates the topics treated in the study from a phenomenological perspective follows.

## The Concept of a Miracle From Different Perspectives

According to the Oxford English Dictionary (2010), a miracle can be understood both as an uncommon act or incident inexplicable by natural laws, and also as a formidable and stupendous event. This ordinary meaning of miracle tends to be commonly employed in the contexts of the health sciences. For example, when a patient hopes for a cure of a terminal disease, and the medical team evaluates this as a total lack of technical foundation (Pinto and Falcão, 2014), the belief in miracle is considered to be something very negative.

But in the philosophical sense, as Swiezynski (2012) explains, a miracle can be understood as an extraordinary event for disagreeing with the prevailing knowledge of the world and its regularities, for example, when someone survives a plane crash thousands of kilometers high or survives an extremely advanced state of cancer. This way of defining a miracle, different from the other, offers a space for mystery, something that eludes the technical knowledge acquired by humans. In this sense, to believe in the miracle of cure would not be seen necessarily as negative, but as a kind of openness to possibilities yet unknown and explained by the present state of medical science.

In the religious sense, miracle refers not only to an extraordinary event but also, and essentially, to the fact of resulting from divine providence or some force that transcends matter or mere human action (Swiezynski, 2012). In this case, it could be seen by the medical team as something positive or negative depending not only on the way this belief is handled by the patient but also and necessarily: (a) on the openness of the health professionals to alternative ways of explaining and managing the processes of health and illness different from the technical knowledge that has been made available by medical science; (b) on their favorable or unfavorable attitudes to the dimension of religiosity and spirituality and its role in people’s lives.

As the phenomenologist Tillich (1967) pointed out, even though the usual definition of a miracle is that of a phenomenon that contradicts the laws of nature, the original sense of the term refers to that which provokes amazement but does not, for that alone, contradict reality. In other words, a miracle is an incident that points to the mystery of being and is an event that is seen as a signal event in an ecstatic experience. Therefore, a miracle would be a revelation of the Divine and not something supernatural (magic). As such, it does not contradict reason, and the sciences, psychology, and history can assist theology in the process of the revelation of a miracle.

In other words, Tillich (1967) criticizes the use of the word miracle to designate something irrational and posits that “Miracles cannot be interpreted in terms of a supranatural interference in natural processes. If such an interpretation were true, the manifestation of the ground of being would destroy the structure of being” (p. 129). According to him, a miracle is part of a structure grounded on reality: “A genuine miracle is first of all an event which is astonishing, unusual, shaking, without contradicting the rational structure of reality (.) One can say that ecstasy is the miracle of the mind and that miracle is the ecstasy of reality” (Tillich, 1967, p. 117). Thus, ecstasy presents both a psychological and transcendent character and reveals a relation between mystery (miracle from an objective point of view) and being: “Ecstasy is the form in which that which concerns us unconditionally manifests itself within the whole of our psychological conditions” (Tillich, 1967, p. 113).

Also, for Saint Thomas Aquinas (1265–1273), a miracle does not necessarily mean something supernatural or something without any contextualization in the real world:

“The word miracle is derived from admiration, which arises when an effect is manifest, whereas its cause is hidden; as when a man sees an eclipse without knowing its cause, as the Philosopher says in the beginning of his Metaphysics. Now the cause of a manifest effect may be known to one, but unknown to others. Wherefore a thing is wonderful to one man, and not at all to others: as an eclipse is to a rustic, but not to an astronomer. Now a miracle is so called as being full of wonder; as having a cause absolutely hidden from all: and this cause is God. Wherefore those things which God does outside those causes which we know, are called miracles.” (Saint Thomas Aquinas, 1265–1273, p. 692).

It can be observed that from the perspectives presented hitherto, only the first definition, taken from a common dictionary, sees a miracle as a necessarily supernatural phenomenon and favors its interpretation as something that has negative behavioral results in health contexts. In this sense, when used as SRC, the hope in a miracle would represent a negation of reality or a blind and passive hope of cure which often leads to a rejection of treatment or prescribed medication as can be verified in different reports of health professionals about the role of religiosity on physical and mental health (Pinto and Falcão, 2014; Freitas, 2020). However, understood in the philosophical or Christian-theological sense, a miracle would not go against natural laws. Instead, it would be an event yet to be explained by the level of scientific knowledge so far attained by humans or by a particular sphere of knowledge. In this frame of mind, the act of praying for a miracle would not necessarily mean a negation of reality, but rather, recognition of the limits of knowledge so far attained by medicine, for example. This view paves the way for the belief in miracles to be seen as also having positive results for health as can be seen in many other Brazilian and international studies (Borges et al., 2015; Carlsson et al., 2017; Borges and Petean, 2018).

On the other hand, still from the conceptual point of view, it is relevant to reflect on the terms used in the original RCOPE scale (Pargament et al., 2000) and its posterior translation for Brazil (Panzini and Bandeira, 2005). This specific modality of SRC is evaluated in this scale through a positive or negative response for the item “I prayed for a miracle.” We should consider the fact that responding affirmatively to this question, the respondent does not necessarily desire cure exclusively. After all, the act of praying is more than supplication and can also be understood as a way of connecting and communicating with God, as Espirito Santo (2016, p. 576) points out:

“Prayer is an intense struggle of a being in the effort to reconnect with the fount of life and meaning which is God. In the innermost realm of being, words lose their meaning and value, this is the moment when contemplation, as metalanguage, becomes a means of communication between human being and God.”

The act of praying for a miracle may present various and complex aspects when observed from a qualitative phenomenological point of view because, in this perspective, what is more relevant is the lived phenomenon grasped in the act (Amatuzzi, 2003). In other words, in the specific case that we address here, what is most important is how the desire or hope in miracle really impacts the lives of patients or their family members, which includes their psyche (emotions and cognition), their behavior, relationship with the world, and all that it entails (including the health professional that cares for them, medication, treatment, and so on). That is to say, what is decisive in evaluating the positive or negative impact on patients’ health would not be the simple fact of their affirming that they “prayed for a miracle” *per se*, but its impact on the life of the patient with grave illness. This would imply discarding conceptions previously undermined by dichotomous interpretations.

Having presented this short conceptual and terminological consideration, we address the various psychological aspects of a miracle and its multiple facets, which should be considered in the evaluation of their positive or negative impact on people’s health.

## Belief in Miracles under Multiple Aspects and Their Different Facets

One of the psychological aspects through which belief can be analyzed, from a cognitive point of view, relates to the locus of control, through the attribution of causality, by which people perceive that life events are internally controlled – e.g., by itself - or externally – e.g., by other agents. Originally, the concept of locus of control was developed by Rotter (1990) to describe how the individual perceives that he or she has or does not have control of his or her life. According to him:

“Internal versus external control refers to the degree to which persons expect that a reinforcement or an outcome of their behavior is contingent on their own behavior or personal characteristics versus the degree to which persons expect that the reinforcement or outcome is a function of chance, luck, or fate, is under the control of powerful others, or is simply unpredictable” (Rotter, 1990, p. 489).

Hayward et al. (2016, p. 888) affirmed that “Religious beliefs may have a number of important implications for one’s health locus of control, and these implications may vary depending upon the specific nature of those beliefs.” Therefore, when religious belief fosters an active stance of care for health in the individual (control mediated by God), the results tend to be beneficial. However, when the belief delegates the function of caring for the individual to the Divine, the results may be harmful. From this point of view, the hope in a miracle, much more frequent in critical situations (Hayward et al., 2016; Borges and Petean, 2018; Bibler et al., 2020), is considered an external and passive way of religious control which would bring harm to the health of the individual. On the other hand, the above authors emphasize that, in end-of-life situations or cases of incurable diseases, delegating destiny to God could often mean acceptance of the outcome and avoidance of the prolongation of invasive and unsuccessful treatment. In the same way, Pargament et al. (1988) affirmed that passive ways of coping are appropriate in situations beyond the control of the individual such as death, terminal illnesses, and accidents, granted that he or she finds protection from anxiety and a haven in religiosity and spirituality, which are important elements for coping with problems whose solutions are beyond his or her reach and that of the health professionals. Such acts as praying for a miracle and leaving the solution “in the hands of God” often emerge in these situations.

However, it is worth noting here that even from the religious point of view, hope in a miracle or the prospect of a miracle does not necessarily imply a merely passive attitude whereby the locus of control is necessarily attributed only to external agents. For example, in the Judeo-Christian theological understanding of miracles, the active participation of the patient in the therapeutic process is presumed. Such participation stems from the hope and optimism that impels the patient to collaborate with the therapist and comply with his or her instructions, not merely waiting passively for a miracle to happen as if it were magic. The paradigmatic case of the cure of Naaman the leper in the Old Testament is a good example here. Elijah the prophet instructs the leper to go to River Jordan and bathe seven times there. The leper, though reluctant at first, heeded the instruction and the miracle occurred (Green, 1986b, 1–14). We find another paradigmatic passage in the New Testament where ten lepers seek Jesus for a cure. He gives them an instruction which they carried out. Upon heeding the instruction, the miracle of cure happened (Green, 1986a, 11–19).

Another psychological aspect that needs to be evaluated when we situationally analyze the role of the belief in miracle in health settings is with regard to its concrete effect on the behavior of the individual and its respective impact on the treatment and the relationship with health professionals. Some authors, specifically concerned with such aspects, discovered patterns and developed classifications for the behaviors of patients and caretakers based on how the latter is affected by their respective beliefs in a miracle. For example, Shinall et al. (2018), based on their studies with adult patients under palliative care, differentiated belief in miracle into four patterns: (a) harmless: when the patient hopes for a plausible positive but improbable result for a cure. This does not generally spark off conflicts with health professionals; (b) Shattered hopes: when faith is paralyzed due to an unfavorable clinical evolution. Generally, this does not generate conflicts with doctors but leads to an important existential pain and impairs the patient’s quality of life; (c) Integrated: grounded on religious dogmas, and may not be in compliance with health professionals, and spark off conflicts in the doctor-patient relationship; (d) Strategic: religion imposes itself on the situation and obstructs wider consultations about care decisions and constitute a negation of reality.

Bibler et al. (2020) recently described a new classification of belief in miracles for caregivers of gravely ill children in the following way: (1) integrated: patients see the clinical state from a religious standpoint and bring religious objects, and may spark off a confrontation with science; (2) Procurators: the child’s caregivers do not depend totally on the religious community and the miracle may assume other meanings beside cure, for example, the well-being of the child; (3) Adaptable: they manifest the feature of having faith but adapt to religions, do not like to talk about miracles, generally, and see the care given to patients with distrust.

Even though the classifications presented above are based on the concern of identifying the beneficial or harmful impacts of the belief in miracles on the patients’ health and the respective treatment, they are not necessarily dichotomic. They are inspired by the concept of coping as a strategy of psychological adaptation; they consider the specific role of belief in miracles and the respective impacts on the medical team and on the patients/caregivers; they take opposite outcomes into account or conciliation and possible psychical consequences of the hope in a miracle that may be harmful to the individual – for example, the cases of shattered hopes, when the miracle does not occur, which impairs the quality of life of the patient; besides the strategic and adaptable cases, where the belief in a miracle leads to conflicts with the medical team since the patient withdraws the locus of control about the illness from the hands of the medical team and transfers it to religion.

Another psychological aspect adopted by the cognitive sciences to evaluate the individual’s psychological adaptation has to do with the type of coping strategy used in times of stress or crises. Folkaman (1984) classified coping strategies in modalities, defined by behaviors or mindset used to cope with stressful events; they are focused on emotion or the problem and maybe concomitantly employed, one supporting the other in specific stressful situations. A coping that is focused on the problem aims at a resignification or a direct action on the event that triggered the stress and tries to modify it either by employing internal (redefinition of the stressful element) or external (negotiate, seek support) actions. But coping focused on emotion is an effort to regulate the emotional state associated with the stress. The efforts are directed to the somatic or psychic level to modify the emotional state of the individual in the attempt to reduce the unpleasant physical sensation of stress. The act of praying for a miracle can also be considered from these two perspectives.

In a certain way, some psychological theories created the stereotype of religion as a coping strategy focused on emotion, representing a defense mechanism (Pargament et al., 2005), thereby concluding that such kind of confrontation may lead to negative psychological adaptations since the individual does not act on the problem by seeking solutions or alternatives of modifying the lived reality (Paiva, 1998). Seen from this stereotyped angle, the desire for a miracle will be easily interpreted as a negation of reality for not focusing concretely on the problem but rather, on pure emotion, as a way of controlling the suffering sparked by the situation of crisis.

It is pertinent here to problematize this dichotomy. After all, the attitude of coping with reality by focusing on emotion does not necessarily mean a negation. Instead, it could mean an attitude of confidence in the future and a hopeful stance – and even resilience, for example, in the face of illnesses of high lethality and grave pain. In various terminal clinical situations, like cancer and neurodegenerative illnesses, there is no solution or way out of the problem through expertise or available medical technology, thereby making it necessary to have recourse to emotion, seeking resilience and acceptance. In these cases, the openness to the unknown, to the existential mystery is oftentimes the best way out since there is no solution within the reach of the patient or the medical team. Even when it appears to be a sheer negation, the hope in a miracle can still fulfill the purpose of “gaining” some internal time to accept the reality of a bleak diagnosis or inevitable death as the studies of Kübler-Ross (1969) point out. From the emotional point of view, this necessity is justifiable and what frequently happens is that the health professional does not have the time or necessary skills to manage this interim between the first reaction of a (de)generative character and its posterior outcomes.

Another example that illustrates the relativity of the judgment of whether SRC strategy focused on emotion is positive or negative, and which also points to the necessity of considering the complex relations between the act of hoping for a miracle and psychological adaptation, is the result of a meta-analysis made by Ano and Vasconcelles (2005). On establishing relationships between the SRC and the psychological adaptation of the individual in the face of a stressful situation, the authors discovered that even when the miracle does not occur, and this is interpreted by the patient (or family member) as he or she being undeserving of such, or as God’s punishment or abandonment, this does not necessarily lead to depressive and anxiety states. On the contrary, he or she can interpret the fact of the prayer for the miracle not being granted as an opportunity of re-signifying and transforming his or her life: “One explanation for this finding is that, although negative religious coping may be harmful, it does not necessarily prevent people from experiencing positive outcomes.” (Ano and Vasconcelles, 2005, p. 474).

The various considerations made so far point to the necessity of adequately contextualizing the clinical situations that surround the belief or hope in a miracle. This natural complexity of the subject demands a cautious and critical evaluation of the initiatives of studies of nomothetic nature, based on scales that tend to group different SRC strategies as positive or negative – among whom is the hope in a miracle, from their previous identification of being: (a) passive or active, in the measure that the locus of control is situated within or outside of the individual; (b) focused on the problem or emotions. So much so that the creators of the RCOPE (Pargament et al., 2000, p. 521) recognize the complexity of the perspective of religion and spirituality in the health sciences: “We recognize, however, that any form of religious coping may serve more than one purpose. Thus, we did not expect to find five factors of religious coping that correspond to these five religious functions.”

Specifically addressing the complexity of belief in miracle, Panzini (2004), also, on translating and validating the SRC scale, described the item “I prayed for a miracle” as ambiguous and recognizes that this item contains both positive and negative aspects and as such not possible to be classified *a priori* in one polarity or the other. Even though no reasons were given for such, the item referred to was not even included in the shorter versions of the RCOPE scale (Pargament et al., 2011; Esperandio et al., 2018), one of them being elaborated/adapted by the same author. Such a version was considered more adequate for research by Vallada et al. (2013) in comparison to the original.

It can therefore be seen that the generalization of the positive-negative dichotomy developed around the belief in miracle is very superficial and does not necessarily favor an understanding of how the belief can be beneficial or harmful to the psychological adaptation of patients and caregivers. This is still true in the case of a country with a high religious population like Brazil.

## Example of Two Studies Carried Out in the Brazilian Context

Brazil has a population of approximately 214 million people (Brasil, 2021) and is one of the twelve most religious countries of the world and the second most religious country in Latin America according to a survey conducted by Win/Gallup International (2015). According to this same survey, 79% of people in Brazil consider themselves religious and 81% consider that religiosity has a positive role in their country. Despite the significant predominance of the Christian religion, according to the results of the last demographic census carried out in the country (Brasil, 2010), there are more than fifteen kinds of religion with Catholicism as the majority (64%) followed by the evangelicals (22.2%). The spiritists officially represent about 3% of the population and the Afro-Brazilian religions are included in this category. Nevertheless, this diversity of religious dogmas and doctrines does not prevent people from transiting between various religions (Mota et al., 2012), and in daily life, many Catholics attend the spiritist religions and vice versa. Steil (2001, p. 124) observes that the present religious miscegenation in Brazil stems from an attitude that is typical of Brazilians: instead of isolating themselves in dogmas, they prefer to seek an “affective authenticity in incorporated spiritual experiences.”

Even though the psychology of religion has been very productive in the last decades, we observe a predominance of qualitative studies (Paiva and Freitas, 2019), and the increase of quantitative studies are more recent (Esperandio and August, 2017). Various scales that evaluate the role of religiosity and spirituality in peoples’ lives have been carried out and validated in the country (Corrêa et al., 2016), e.g., the module of religiosity and spirituality in the instrument of the evaluation of the quality of life -WHOQOL-SRPB (Panzini et al., 2011), Intrinsic Religiousness Inventory (Taunay et al., 2012), Spiritual Well-Being Scale (Marques et al., 2009), Multidimensional Measurement of religiousness/Spirituality (Curcio et al., 2013), Duke Religion Index (Lucchetti et al., 2012), RCOPE (Panzini, 2004; Panzini and Bandeira, 2005), and Brief Scale for Spiritual/Religious Coping (Esperandio et al., 2018). The only scale, among all, that considers the belief in a miracle is the RCOPE, translated, adapted, and validated by Panzini and Bandeira (2005), and originally elaborated by Pargament et al. (2000). Therefore, this is the scale that has been used in Brazilian studies until now to investigate this modality of SRC and its relations with health in quantitative research carried out in the country.

We shall present and discuss two studies carried out in the country about the role of miracle in health contexts to illustrate the importance of the reflections made in this paper. One of the studies was carried out by Leal (2020) and had the aim of evaluating religious-spiritual coping in expectant mothers with fetal congenital malformation (FCM). The study included 99 expectant mothers with FCM of diverse prognoses and 1/3 of the sample having lethal anomalies. The RCOPE scale originally elaborated by Pargament et al. (2000) and later translated and validated for Brazil by Panzini and Bandeira (2005) was applied *in toto*. The results showed that 92.8% of the sample presented positive coping strategies (PSRC), and only 7.2% presented negative coping strategies (NSRC).

Item 45 of the scale referred to, originally developed to evaluate NSRC, examines if the expectant mother prayed for a miracle: “I prayed for a miracle.” It was found that 89% of the expectant mothers (i.e., 88 out of 99) responded affirmatively to this item. Out of this number, 91.2% (81/89) presented PSRC and only 7.8% (7/89) presented NSRC. No socioeconomic, epidemiologic, or clinical condition was statistically significant to identify the profile of the women that prayed for a miracle. However, as we see in Table 1, there was a positive correlation between “pray for a miracle” and all the positive factors of coping (PSRC) – where we find “self-transformation or transformation of one’s life” and “removal of the problem through God, religion, or spirituality.” The correlations between “pray for a miracle” and negative factors of coping (NSRC) were positive for only two of them: “negative attitude before God” and “dissatisfaction with other and/or institution.” Such results point out that the act of praying for a miracle presents significant positive aspects for expectant mothers with FCM, and much less negative ones, in contrast to what would be initially expected from its original classification as NSRC in the scale originally elaborated in the North American context and later translated for Brazil.

**TABLE 1:** Correlation of the factors of Spiritual/Religion Coping (SRC) and praying for a miracle.

Spiritual/Religion Coping	Factor	Spearman (r) correlation	*p*
Positive	Self and/or one’s life transformation	0.51	0.001*
	Actions in search of spiritual help	0.42	0.001*
	Offer of help to other	0.34	0.01*
	Positive stance before God	0.48	0.001*
	Personal search of spiritual growth	0.50	0.001*
	Actions in search of institutional other	0.49	0.001*
	Personal search for spiritual knowledge	0.37	0.001*
	Removal through God, religion or spirituality	0.49	0.001*
Negative	Negative re-evaluation of God	0.11	0.27
	Negative stance before God	0.43	0.001*
	Negative re-evaluation of the meaning	0.14	0.15
	Dissatisfaction with the institutional other	0.21	0.04*

Reproduced from Leal (2020, p. 73). *Statistically significant correlation.

Another study carried out in the Brazilian context also addresses the subject of miracle and aimed at integrating the quantitative and qualitative aspects in two phases in the same study. To evaluate the religious-spiritual coping among family members of children under chemotherapeutic treatment for cancer (Jaramillo, 2019; Jaramillo et al., 2019), they employed the same scale of Pargament et al. (2000) translated by Panzini and Bandeira (2005), for an analysis of nomothetic nature. The second stage was conducted with a study of idiographic nature through interviews with some family members who responded to the RCOPE scale. The first stage counted on the participation of 64 caregivers where an SRC of 3.7 was found, with 3.4 being the medium of PSRC, and two being NSRC. The difference found between PSRC/NSRC was 0.6 which points to the predominance of negative strategies of coping, which was from the point of view of statistical analysis, attributed to the high frequency of the act of praying for a miracle accompanied by a negative stance before God when the miracle does not occur.

In the second stage where interviews were conducted with 14 family members, permitting a qualitative, dynamic, and contextual analysis (Jaramillo, 2019; Jaramillo et al., 2019), it was found that: (a) the belief in a miracle was often associated with biblical readings and this provided more internal control of emotions and afflictions; (b) the belief in a miracle enabled the family members to identify little victories experienced by the child, in such a way that the overcoming of the various stages of treatment could be interpreted as real miracles, making for a constant resignification of experiences, and as such in consonance with the concept of the search for meaning in spirituality. Curiously, even when the miracle that was experienced as a manifestation of hope and resignification, is manifest in the speeches of patients and caregivers, based on their analyses of a more quantitative nature, the authors maintained the interpretation about the “act of praying for a miracle” as an NSRC strategy, justifying that the prayer for a miracle occurs due to the fear of death and its respective negation.

## Discussion and Conclusion

Various aspects need consideration in understanding the results of the studies referred to above. We must emphasize here the risks of a previously established classification including the item “I prayed for a miracle” in the group of strategies of NSRC independent of any cultural and situational contextualization. It was seen in the first study that this item was much more correlated positively with the other items classified in the group of the PSRC than the set of the NSRC. And in the second study, it was seen that the results of qualitative nature showed various positive impacts of the act of praying for a miracle in the perception of the interviewees. The bias of an early classification that negatively connotes the belief in a miracle ignores all the multiplicity of its aspects and facets that were previously mentioned in sub item 3 of this study. It reveals the danger of a nomothetic evaluation that promotes a kind of crystallization of the negative interpretation of the act of praying for a miracle. This danger is more poignant when it substitutes a more effective effort of seeking to understand and qualify the way this experience is lived by patients or caregivers, considering in-depth the existential and sociocultural aspects related to such experiences.

In the first study referred to, even though it did not include a second stage, of a qualitative and idiographic nature (Leal, 2020), it sought to reflect on the results found and raises the hypothesis that in a certain way, for expectant mothers with malformed fetuses, the belief in miracle can be, in a certain manner, a way of warding off reality but without necessarily negating it, enhancing a kind of continuum from the prenatal to the puerperium where hope can be nurtured; so that birth and first care of the baby may be less distressing. And in the process, make possible a resignification of life in cases of lethal FCM where the expectant mother may recognize to have exercised maternity, be it for a short time (Leal, 2020). Or still, under another aspect, the experience of “praying for a miracle” occurs initially as an act of openness to life instead of the immediate closure of a bleak perspective imposed by a technical and scientific perspective. This openness is sound from the psychological and existential point of view in so far as through it, there is also the time necessary to confront the realities of FCM.

In the second study, even though they included a second stage of qualitative nature, the authors (Jaramillo, 2019; Jaramillo et al., 2019) ended up giving more emphasis to the results obtained from the scale and accentuating the negative interpretation of the act of praying for a miracle. The danger in this, from an epistemological point of view, is reinforcing a kind of orthodox faith in science that attempts to classify and quantify, and in this specific case, generalize the principle that coping focused on emotion is always negative. Another danger, now from the practical point of view, is reinforcing a dichotomy between positive and negative, which can create a great barrier in the relationship between health professionals and patients, by stigmatizing the act of praying for a miracle and always taking it as a desire for something supernatural in detriment to medical knowledge. The professional entangled in this dichotomy may fail to see clinical aspects essential to the quality of his or her care of the patient or family members. He or she may, for example, be completely blind to one of the aspects pointed out by Delisser (2009): the caregiver or patient, on referring to his or her desire for a miracle, maybe communicating both the wish of being an optimist and hopeful in the attempt of maintaining a positive attitude in the face of a grave illness, and also the sentiments of anger, frustration, or disappointment with the care given by the medical team.

It should be admitted, therefore, that the classificatory questionnaires do not encompass all the complexity of the belief in a miracle and may spark misguided interpretations about what the patient or caregiver is seeking in fact: whether an internal elaboration for better handling of the situation; a temporary delegation until he or she is more strengthened to take charge of his or her responsibilities; a negation of the gravity of his or her illness; or to simply offer a response that confronts consciously or unconsciously the previous conceptions of the health professionals. After all, in the context of the medical sciences, more and more specialized and technical, (Clarke, 2018), a veritable stratification of illnesses and patients’ and caregivers’ behaviors can be verified, based on studies that quantify incidences and prevalence, but without considering how the experience of illness can affect the patient and caregivers. In the specific context of managing the belief in a miracle, this is well illustrated in the study of Green (2015), where it is observed that various nurses in the neonatal intensive care unit tended to be nervous only on the pronunciation of the word “miracle.” These prejudices end up bolstering reactive mechanisms in patients, who may respond antagonistically, preferring to deposit their faith and hope in divine miracle than share with the medical team the same faith that the latter deposit in secularized science (Dzeng and Booth, 2018). In a certain way, such situations remind us of what Grinstead (2018, p. 70) rightly affirms: “While medicine’s emphasis on scientific rigor and evidence-based practice is helpful in many wondrous ways, it must also allow a space for the ineffable qualities of human existence.”

We emphasize, therefore, the necessity of broadening and intensifying qualitative studies (Clarke, 2018) on the effective role of the belief in a miracle, properly contextualized culturally, and which also promotes more articulation between spirituality/religion and the medical sciences, especially when both seek the same end: caring for the human being (Grinstead, 2018). Among the qualitative initiatives, we highlight the contribution of studies of phenomenological nature for offering a wider and deeper vision of the experience lived by patients or caregivers and seeking a convergence of knowledge from other areas of study like theology, psychology, and medicine to better understand the different contexts in which the desire for a miracle is manifest and its most intimate meanings for the individual:

“When clinicians incorporate a basic understanding of phenomenology into their approach to a patient or their surrogate’s resolute insistence to wait for a miracle, this theoretical underpinning may form a foundation for building a mutual understanding with the miracle seeker, as well as a reverence for the inherent mystery of the wide spectrum of human experience” (Grinstead, 2018, p. 70).

In this way, a phenomenological understanding of the belief in a miracle can promote comprehension of the mechanism of religious-spiritual coping beyond a mere dichotomic judgment between “positive” or “negative”, addressing other relevant existential aspects like the degree of subjective and intersubjective flexibility or inflexibility that the individual demonstrates in the face of stress; how the belief in miracle is sustained by a genuine sentiment of spirituality or religious faith; how this faith is sustained by the necessity of attributing the search for meaning to illness or bleak diagnosis, enhancing the hope to manage desolating sentiments like anguish, guilt, or anger of an FCM pregnancy or having a child with cancer independent of a previous classification from the locus of external or internal control or of passive or active attitudes.

“Hope for miracle” for mothers with FCM or family members of children with cancer can be a manner of shying away from reality without denying it, and constitute part of a process of psychic adaptation to suffering and culminating into the search for the meaning of lived experience, and often represented in the peak of resignification through maternal love. It can also be a kind of network support where family members nurture the hope of the patient still under the impact of the sad diagnosis. Thereby, their prayers for a miracle, fortify affective bonds and the reciprocal hospitality between them until they are emotionally more prepared to handle the limits or complete impossibility of reversing the diagnosis. This process can be healthy when it gradually permits an internal elaboration that propels a resignification of the diagnosis in the lives of all the people involved.

Nevertheless, the negative aspect of hope in a miracle is also observed in clinical practice when the expectant mother or family member of the child with cancer (or other bleak diagnoses) insistently seeks the improbable cure, even in the face of medical evidence to the contrary. When such a search is based only on the dogmas that some religious institutions adopt and encourage, remaining static, anchored on a linear interpretation of “miracle,” at the service of traditions and orthodoxy inclined to religious fundamentalism, it can be problematic, closed to the process of resignification over time. In this situation, the locus of external control and the passive attitude is harmful to the psychological adaptation of the patient or of the family member since the focus is not transcendence but on the pragmatic result desired by him or her and then based on a religious doctrine.

The nature of the impact of the belief in miracle turns out to be not only of the accountability of the patient and family members but also of the health professionals. And in this sense, the reports of some professionals in studies of phenomenological nature carried out by Freitas (2021) are paradigmatic. Curiously, when some of them were interviewed about the nature of the relations established between religion and health, based on their clinical experiences, they replied that the relations can be of a positive or negative nature, healthy or not, depending on the approach of each professional and the quality of his or her practice in handling the religiosity of the users of health services. It can be seen from this kind of response that, instead of being simply grounded in the mere dichotomy between science and religion, there is a honest self-accountability of the professional in the process of care of the patient and his or her caregiver. In this process, the health professional takes on the competency and responsibility of not only the strict technical-scientific knowledge, but also the development of cultural and existential skills in the management of complex situations, avoiding the mere underpinning of linear, reductionist, dichotomist, and or hegemonic models.

## Author Contributions

MF conceived the idea of the study and supervised the project. ML and GdM designed the statistical analysis. EN drafted some parts of the manuscript especially those connected with theology, collaboration in the final revision, and translation to English. MF wrote the manuscript with the help of ML and EN. All authors contributed to the final manuscript, each with a specific focus.

## Conflict of Interest

The authors declare that the research was conducted in the absence of any commercial or financial relationships that could be construed as a potential conflict of interest.

## Publisher’s Note

All claims expressed in this article are solely those of the authors and do not necessarily represent those of their affiliated organizations, or those of the publisher, the editors and the reviewers. Any product that may be evaluated in this article, or claim that may be made by its manufacturer, is not guaranteed or endorsed by the publisher.
